# Decision makers’ experience of participatory dynamic simulation modelling: methods for public health policy

**DOI:** 10.1186/s12911-018-0707-6

**Published:** 2018-12-12

**Authors:** Louise Freebairn, Jo-An Atkinson, Paul M. Kelly, Geoff McDonnell, Lucie Rychetnik

**Affiliations:** 1 ACT Health, GPO Box 825, Canberra, ACT 2601 Australia; 2 The Australian Prevention Partnership Centre, Sax Institute, PO Box K617, Haymarket, Sydney, NSW 1240 Australia; 3 School of Medicine, University of Notre Dame, PO Box 944, Broadway, Sydney, NSW 2007 Australia; 4 Decision Analytics, Sax Institute, Sydney, Australia; 5 Sydney Medical School, University of Sydney, Sydney, NSW 2006 Australia; 6 School of Medicine, The Australian National University, ACT, Canberra, 2601 Australia

**Keywords:** Dynamic simulation modelling, Participatory modelling, Public health, Prevention policy, Diabetes in pregnancy, Gestational diabetes, Alcohol, Childhood obesity, Decision support, Multimethod modelling, Hybrid modelling, Knowledge mobilisation

## Abstract

**Background:**

Systems science methods such as dynamic simulation modelling are well suited to address questions about public health policy as they consider the complexity, context and dynamic nature of system-wide behaviours. Advances in technology have led to increased accessibility and interest in systems methods to address complex health policy issues. However, the involvement of policy decision makers in health-related simulation model development has been lacking. Where end-users have been included, there has been limited examination of their experience of the participatory modelling process and their views about the utility of the findings. This paper reports the experience of end-user decision makers, including senior public health policy makers and health service providers, who participated in three participatory simulation modelling for health policy case studies (alcohol related harm, childhood obesity prevention, diabetes in pregnancy), and their perceptions of the value and efficacy of this method in an applied health sector context.

**Methods:**

Semi-structured interviews were conducted with end-user participants from three participatory simulation modelling case studies in Australian real-world policy settings. Interviewees were employees of government agencies with jurisdiction over policy and program decisions and were purposively selected to include perspectives at different stages of model development.

**Results:**

The ‘co-production’ aspect of the participatory approach was highly valued. It was reported as an essential component of building understanding of the modelling process, and thus trust in the model and its outputs as a decision-support tool. The unique benefits of simulation modelling included its capacity to explore interactions of risk factors and combined interventions, and the impact of scaling up interventions. Participants also valued simulating new interventions prior to implementation in the real world, and the comprehensive mapping of evidence and its gaps to prioritise future research. The participatory aspect of simulation modelling was time and resource intensive and therefore most suited to high priority complex topics with contested options for intervening.

**Conclusion:**

These findings highlight the value of a participatory approach to dynamic simulation modelling to support its utility in applied health policy settings.

**Electronic supplementary material:**

The online version of this article (10.1186/s12911-018-0707-6) contains supplementary material, which is available to authorized users.

## Background

Evidence informed decisions are essential to ensure that health policies provide cost effective and high-quality programs and services. However, barriers to the use of evidence to inform decision making remain [1]. Policy and program decision making processes are frequently non-linear and iterative. They are influenced by a range of factors, that compete with research evidence, such as the political environment, budget and resource constraints, and public perceptions of the value of policy options being considered, [1–6]. Evidence provided to policy makers needs to be in a form that is useful and relevant in this context [4, 7, 8]. Policy makers require synthesised and contextualised evidence that establishes the need for a policy response, compares and prioritises policy options, and demonstrates cost-effectiveness of interventions [4, 9].

Research evidence for the prevention of chronic disease often points to a large range of contributing risk factors, including broader social determinants of health [10, 11]. Without tools to make sense of this complex array of evidence it is difficult to understand the dynamic interactions of risk factors and interventions [12], potentially leading to the adoption of approaches that may seem intuitive but fail to deliver reductions in disease burden at the population level or lead to unintended consequences [12, 13].

Systems science methods are well suited to public health and disease prevention questions because the approach takes into account the complexity, context, dynamic nature, and system-wide behaviour associated with public health issues [14]. Dynamic simulation models recreate complex systems and human behaviours as computer simulations. They can be used to synthesise evidence, answer hypothetical questions about the potential outcomes of policy and intervention options, and inform decision making [13, 15].

Incorporating a participatory process into the development of dynamic simulation models can facilitate the exploration of how multiple environmental factors, individual risk profiles and interventions interact [16, 17]. It can be used to enhance knowledge about the focus issue from the perspective of different disciplines, explore conflicting views, test potential solutions to complex issues and even to develop a shared language about the issue which can support ongoing communication [18–21]. The participatory model development process involves an in-depth, interdisciplinary deliberation and co-production process to initially map the causal pathways for the focus issue, and the mechanisms by which interventions have an effect on outcomes [19]. A range of evidence is synthesised, including empirical evidence, expert and practice based knowledge, and theory, to develop, quantify and test a simulation model of the issue [13, 18, 19, 22, 23]. The resulting dynamic simulation model can be used as a decision support tool to explore complex problems such as the prevention of chronic disease and simulate proposed policy and practice scenarios [13, 23].

Advances in technology have led to increased adoption of tools and methods aimed at integrating diverse evidence sources to inform decision making [13, 17]. However, rigorous assessment of the value and utility of these methods and tools is required if their adoption to support evidence informed policy and planning in the health sector is to be achieved. The uptake of dynamic simulation modelling in health has lagged behind other sectors, such as the environmental sciences and business industry [24], and it has been argued that this has, at least in part, been due to limited engagement with stakeholders and involvement of end-users in health-related simulation model development [13, 21, 24, 25]. This has also impacted on the implementation of model findings [24, 26] and led to a reluctance among “non-researchers” to use models as decision support tools [21, 24]. Where policy makers have been included in the simulation modelling process there has been limited examination of their experience; e.g. perspectives on the utility of the model, learning relating to the development and use of the model, or commitment to implement the model findings [13, 27, 28].

This paper reports on the experience of end-user decision makers, including senior policy makers and health service providers, who participated in three participatory simulation modelling case studies in Australian health policy settings. We report on their perceptions of the value and efficacy of this method as a tool for evidence synthesis and decision support in applied health sector policy and service planning contexts.

## Methods

### Context

The Australian Prevention Partnership Centre (http://preventioncentre.org.au/), in collaboration with jurisdictional governments have pioneered the co-production of sophisticated, multiscale dynamic simulation models to support health policy and practice decisions [29–33]. In developing these models, researchers partnered with health departments, clinicians and regional planners in collaboration with a multidisciplinary group of stakeholders using a participatory process [32, 33]. This research is based on three of these case studies described briefly below (Table 1). The case studies and participatory processes are described more fully elsewhere [34].

**Table 1:** Description of dynamic simulation modelling case studies and context

Topic area	Type of model	Model development period	Context	Application to decision making
Reduction of alcohol-related harms (Alcohol)	Agent based model	2015–2016	Alcohol misuse is an important public health issue for which there are complex causal mechanisms and contested intervention options. This model was developed to inform jurisdictional government strategies for reducing alcohol-related harms.	The model represents the heterogeneity of alcohol use across the population, how the dynamics of drinking behaviours vary across the life course, the harms, both short and long term, that arise from alcohol use, and the differential effects of interventions across subgroups in the population.
Reduction of childhood overweight and obesity (Obesity)	System dynamics model	2016	In 2015, an Australian State Premier set an ambitious target to reduce childhood overweight and obesity in children by 5 % over 10 years. It was predicted that additional strategies, or combinations of strategies, would be required to achieve the Premier’s target. Decision makers were presented with the challenge of determining where best to focus resources and efforts.	The model explores the complex issue of child overweight and obesity, incorporates existing programs and tests the likely impacts of a range of policies and programs. It forecasts the combination of interventions required to achieve the Premier’s target.
Prevention and management of Diabetes in Pregnancy (DIP)	Hybrid model (system dynamics, agent based modelling and discrete event simulation)	2016–2017	. Diabetes in pregnancy is increasing in Australia and internationally and exploration of new strategies to prevent and manage the condition is needed. The model considers the short, and long-term implications of the increasing prevalence of both DIP and associated risk factors.	The model focuses on the development of Diabetes in Pregnancy (DIP) from the perspective of the individual. Prevention interventions were prioritised in the model as delays in the development of diabetes will potentially result in reduction in the longer-term burden of disease and costs to the health system. However, the model can also explore clinical interventions. Health service utilisation has been captured in the model enabling it to explore the resource impact of model of care scenarios.

### Procedure

The evaluation of the participatory modelling process was informed by the Challenge and Reconstruct Learning (CHARL) Framework proposed by Smajgl and Ward [35]. The CHaRL framework involves assessing formalised and facilitated learning among decision makers and decision influencers at varied policy levels in deliberative processes. The key component of the CHaRL framework is the change in perception or belief about assumed causality within the system. The change in perceptions or beliefs can be measured using individual value and attitude/belief orientations recorded by participants before and after the modelling process [35].

The three modelling case studies were chosen to allow for data to be collected at all stages of the participatory process. The model development process had been finalised in two of the case studies, and data were also collected on the use of models to inform decision making. Data collections are described below.

For data triangulation, key informant interviews and participant observation during workshops (field notes kept by lead author LF) were used to collect information across the three case studies as outlined below.

1. Pre-workshop interviews (Diabetes in Pregnancy project *n* = 5)
2. Post workshop interviews (all case studies *n* = 7)
3. Workshop observations (all case studies, total workshops *n* = 9)

Qualitative analysis was conducted for the transcripts from the semi-structured interviews, and the observation field notes. The data collection and analysis methods are described in detail below.

### Participants and sampling

Purposive sampling was used for each case study to recruit participants with a range of expertise, including providing or planning health services, undertaking research or developing policy for the issue in focus. Pre-process interviews in the DIP case study occurred with six participants that included senior clinical and public health policy decision makers.

Sampling for the post workshop interviews (*n* = 7) ensured representation across the three case studies to include perspectives from case studies with models at different stages of development and project roles, (e.g. facilitator or participant), or policy making roles, (e.g. clinical, policy, or public health executive). Some interviewees had participated in more than one case study. Focus for selection in the post-process interviews was on the setting where policy change decisions would occur i.e. interviews were with participants who were employed within government agencies with jurisdiction over the relevant policy decisions. Recruitment of interviewees continued until saturation was reached for the main themes and categories.

### Data collection

Participant experiences and perspectives across all three case studies were collected in semi-structured interviews with key informants focusing on their personal response to the participatory modelling process and perceptions of the:

- ○ value of simulation modelling as policy decision support tool
- ○ strengths and limitations of the method and intention to use simulation modelling in the future
- ○ perceived enablers and barriers to the use of simulation modelling

Interviews were conducted face to face where possible (*n* = 5), however telephone and web conferencing interviews (*n* = 8) were also used to allow interviews with participants in distant locations. Interviews across formats were of comparable length (ranging from 30 to 60 min) and depth of exploration of the issues discussed. Interviews were conducted by the lead author and a research officer (EO - two pre-workshop interviews, see acknowledgements). Indicative questions for pre- and post- interviews are presented in Additional file 1. Field notes were based on observations of the participatory workshops and debriefing discussions between the authors and research officers (EO, NR and CW, see acknowledgements).

### Data coding and analysis

Interviews were audio recorded and transcribed using a professional transcription company, checked for quality and de-identified. The transcriptions were coded and analysed by LF. The analysis was guided by grounded theory principles [36, 37]. An iterative process of descriptive coding and analytical memos was used to develop themes and conceptual categories, explore their inter-relationships, and to triangulate insights from the interview and field note data. The progressive analysis was iteratively reviewed by JA and LR and further revised as new data was incorporated. Analytic memos written by LF were also shared with JA and LR to facilitate the analysis review process.

## Results

Table 2 provides an indication of the data analysed for each results section.

**Table 2:** Overview of data analysed for each results section

Results section	Analysis based on:			
	Pre-modelling interviews	Post-modelling interviews		
	DIP	DIP	Alcohol	Childhood Obesity
Pre-modelling perceptions of evidence use in decision making	X			
Experiences of participatory modelling		X	X	X
Learning through participation		X	X	X
Experience of using dynamic simulation modelling to inform decision making			X	X

*DIP* - Dynamic simulation modelling to inform the prevention and management of diabetes in pregnancy

*Alcohol* - Dynamic simulation modelling to inform reductions of alcohol related harms

*Childhood Obesity* - Dynamic simulation modelling to inform how best to reach the Premiers target for reducing childhood overweight and obesity

### Pre-modelling perceptions of evidence use in decision making

Prior to the commencement of the participatory modelling process, respondents consistently emphasised the importance of evidence-informed decision making however, they identified challenges relating to the availability, applicability, persuasiveness, timeliness and accessibility of evidence to inform decision making.

Lack of evidence was described as the “biggest challenge” in circumstances where no policy relevant evidence existed, or the available evidence was not sufficiently robust to inform health policy or service decisions. In situations where rigorous studies like randomised controlled trials were not likely to be conducted due to ethical, practical, or funding constraints, there was a clear view that an ongoing lack of evidence was unlikely to be resolved using traditional methods of research. Further, when evidence was available, it was not necessarily applicable to local health service or population context, making it difficult to use for local policy decisions.

Evidence was reported to be only one of many competing factors involved in decision making and respondents described other factors as more powerful decision influencers. Evidence needed to be convincing to compete with these other influences, including the input of advocacy groups, incentives and restrictions built into funding models and internal (organisational) and external (political and community) competition for resources.

It was also reported that the use of evidence in policy decision making continued to be limited by poor accessibility. Thus, research needed to be communicated in more accessible ways to policy makers who vary in their level of expertise in interpreting and applying research findings. Policy makers and program planners often prioritised government reports and statistics, and non-government organisation reports to inform decisions as they used more accessible language and were free to access. Some policy makers were unable to access journal articles behind a “pay wall”. It was widely reported that there was little time in policy settings to explore evidence in detail or to conduct research. In this context, respondents consistently identified that there was significant room for improvement in the way evidence was translated and used to inform policy and practice decisions. As a result, respondents were motivated to explore new methods, such as participatory dynamic simulation modelling, to see how they could improve and increase the use of evidence in their applied settings.

### Experiences of the participatory simulation modelling process

As identified in Table 2, this section is based on post-modelling interviews across the Diabetes in Pregnancy, Alcohol and Childhood overweight and obesity case studies.

#### Motivations for participating

Due to the significant time investment involved in participatory dynamic simulation model development, the opportunity costs and likely outcomes from the modelling projects were significant factors in policy makers’ decision to participate in the process. Targeted and tailored engagement, facilitated by a trusted domain expert, with key participants in the planning phase of the project was important to justify the benefits of participating and ensure that from the policy makers’ perspective, the topic was high priority, of professional interest, complex and had contested options for intervening. Across the modelling case studies, many interviewees reported that they explicitly considered the opportunity cost of participating in the dynamic simulation modelling project before deciding to become involved. They went through a process of weighing up whether the likely outcomes from the modelling projects would be worthwhile given the significant time commitment required.

Commonly reported reasons for agreeing to participate included: the person’s professional involvement and expertise in the topic being modelled, the trusted relationship with either the modelling team or the lead domain expert involved in the project, curiosity about dynamic simulation modelling and participatory methods, and aspirations to improve the use of evidence in decision making. These are explored further below. A few participants were directed to participate by their organisation.

The choice of focus topic to be modelled was a key criterion for agreeing to join the participatory dynamic simulation modelling project. The topic needed to be an important local priority of current professional and or organisational concern with complex causal risk factors and need for policy or programme response. In order to justify the commitment to participate the topic needed to be complex with different perspectives of which causal factors and exposures were important, different intervention options to decide between, contested views about what works and what doesn’t, and where the combined impact of interventions was unknown.

Trust in and familiarity with the project team were commonly identified as important factors influencing the decision to participate. Most interviewees embarked on the project with little or no experience of dynamic simulation modelling, however they had trust in the modelling team or domain expert who initially approached them, and this facilitated their decision to participate.

“It's having known [facilitator] for a long time through the [work area], and our work with the [topic X]. [Facilitator] knowing that part of my research was based about [topic X] disease, and various other things I've been looking at.” (Senior clinician)

An interest in learning new methods to facilitate evidence informed decisions in public health was commonly identified as a reason to participate in the case studies.

“I was interested in processes for better being able to inform and educate the policy making process” (Public health executive)

“it’s an incredibly useful policy tool if it's done appropriately with the right people asking the right questions. It's a very powerful tool.” (Public health executive)

Goals for participating in the case studies included learning about the process of developing a simulation model and how it can be used to inform decision making. The participatory approach was highly valued by most interviewees as it provided an opportunity to combine their expertise with the expertise of the modelling team to produce an innovative decision support tool.

#### Engaging in the participatory activities

Workshop participants engaged in a range of large and small group activities to collaboratively develop a conceptual map of the problem, prioritise and map the impact of interventions to model architecture and identify data and evidence to incorporate into (or parameterise) the models. These activities have been described in detail elsewhere [34].

#### Collaboration and co-production

Collaboration and co-production were identified by participants in these case studies as the unique and highly valued aspect of the participatory dynamic simulation model development. The participatory approach facilitated the contribution and synthesis of a significant knowledge base and was critical to eliciting and negotiating priority causal factors, exposures and interventions to be represented in the model. The process of contributing expertise and then explicitly seeing how it was used in the model were important factors in facilitating engagement and a sense of ownership in the model.

“The session with lots of string and sticky notes and things on the board, it looked a mess and going away thinking, “How are they going to use all that?” But I actually was surprised, pleasantly surprised, at how that was actually used to inform the development of the model, and it really was.” (Senior clinician)

The contribution of considerable knowledge and diverse expertise of participants was consistently identified as important. Many respondents commented that they were surprised at the willingness of participants to contribute their experience, ideas and knowledge to educate the modelling team about the issue and guide the model development.

“I think the participatory approach, you're having people in the room that have accumulated knowledge, expertise in the area over quite a number of years, actually brings a lot of knowledge into that room, and it's not possible for one or three or five people to do the literature searches and understand all the information” (Senior clinician)

Respondents also noted that people “put their egos aside” during the workshops as the interdisciplinary and co-production approach meant that participants were learning from each other. The content expert participants learned about dynamic simulation modelling and the modellers learned about the priority public health issue being modelled.

While being time consuming and personally challenging for some respondents to engage in; the interactive activities and group discussions were viewed as critical to eliciting and negotiating priority causal factors, exposures and interventions to be represented in the model. Many respondents noted that the same outcome could not be achieved through one on one discussions as the inclusion of a diverse range of participant perspectives was important to guide the model development.

“By having everybody in the same room, you got to really be able to relate to everybody's little piece of the puzzle. I think if you had just done that with individuals, you wouldn't have got the model that you've developed.” (Senior clinician)

The lack of consumer involvement in the modelling case studies described in this paper was identified as a gap in representation. However, respondents also noted that finding consumer representatives and strategies to realise the benefits of consumer input can be challenging in some circumstances e.g. when the discussion is focused on highly specialised biological causal mechanisms of disease development and progression.

Some respondents reported that they initially perceived the workshop activities to support active participation as less rigorous or evidence-based than they had expected. For example, that some participants were relying on their opinion rather than evidence, were advocating for causes or had a priori preferences for particular actions and thus a potentially biased view of the evidence. A couple of participants noted that they observed differential engagement by other participants with the activities and “people not taking it as seriously as perhaps they should have”. However, most of these respondents also noted that as the model development process progressed they were reassured by the use of good data and evidence to inform the model and to test hypotheses that came from expert opinion.

“I was a bit sceptical of that process, and in terms of input given that it's meant to be evidence-based inputs through that conceptual mapping of the bits of paper and string, and plaster, ... I expected it to be more rigorous, but I learned by doing it, that really it was more about informing the modelling team in terms of logic, structure and models, and then they went away and found the evidence, if you like, to support the link of this, and of course all pathways, and the association pathways.” (Public health executive)

Contributing expertise and then explicitly seeing how it was used in the model were important workshop activities. The process of unfurling the model by describing the logic and architecture and relating it to discussions at previous workshops was highly regarded by participants. The model was viewed as “the fruits of their labour” and a sense of innovation and excitement was expressed by many respondents.

Two key roles were identified as facilitating engagement in these case studies. The first was having a trusted domain expert for each project who was a well-respected authority on the focus issue and played a lead role in the project planning and workshop facilitation. The domain expert facilitated the approaches to key stakeholders, increasing the likelihood of their agreement to participate, and was a known colleague to promote engagement in the participatory process. The second “translator” role was identified by respondents who were more closely involved in facilitating workshops and working with participants. This role involved both explaining the policy context, e.g. current policy priorities and interventions, and contextualising the data, e.g. explaining data collection methods, representativeness and limitations, for the modelling team and conversely translating the model requirements and development process to the workshop participants.

Having an embedded policy officer, who along with the lead domain expert, played the role of translator working within the modelling team ensured that the model was policy relevant, well understood and used. A key aspect of this was being able to run model scenarios independently of the modelling team to provide timely responses to policy questions (e.g. switching interventions on and off and/or modifying parameters such as reach and effectiveness and producing results).

#### Participant learning through group model building

Respondents reported that the participatory process worked well in facilitating interactions and contributing expertise. The process also provided an opportunity for the expert participants to be exposed to multiple perspectives and frameworks for viewing the problem but was not identified as resulting in individual learning about the focus issue. A commonly cited reason for this was the selection of participants who were experts in the focus issue, many of whom had dedicated their career to working on it. They came to the process with a good understanding of the different perspectives and complex causal relationships e.g. costs, drivers, and evidence-based strategies.

“I think it gave people a broad picture and they recognised where everybody's different areas fit. But whether it actually changed how they link things together, I don't know.” (Senior clinician)

However, all respondents reported that they learned about the potential of dynamic simulation modelling to support decision making and the process of developing simulation models through the participatory process.

“What we learned is the potential value of developing a simulation model. Talking to the others, everyone was quite impressed by where it's got to, so the exchange and knowledge between the different discipline areas, I guess, was the most positive. ... I don't think you've changed our views very much on [topic X]. ” (Senior clinician)

Interviewees identified that the participatory modelling process and model outputs allowed participants to develop insights into the interrelationships between causal factors and emergent behaviour of the system i.e. “If you change your practice how does that impact on other parts of the system”; explore the combined impact and interaction between interventions; and explore new and untested interventions in the model prior to them being implemented in the real world. These important learning outcomes and how they can be used to facilitate policy conversations are discussed in more detail below.

“That's I think what the benefit of the model will be, is that it can show to people if you change one thing here, what's it going to change for everything else.” (Senior clinician).

### How dynamic simulation models facilitate the use of evidence to inform decision making

The following analysis is primarily based on responses regarding the two simulation models that were finalised at the time of writing (Alcohol and Childhood obesity). The exception to this is the discussion regarding the identification of evidence gaps which includes responses from all case studies.

#### Participatory approach and trust in the model as a decision support tool

The participatory approach used in the model development engaged respondents actively in the process and increased their familiarity and trust in the model outputs. Respondents reported an increased sense of ownership and interest with these models compared with other modelling projects that had not used a participatory approach.

“…there were times where I'm thinking, “Really? We know all this stuff, and do we have to spend all this time?” But you realise that that's the nature of it, that if we didn't go through those processes there wouldn't be the same trustworthiness, or people wouldn't trust it as much, they'd be questioning it.” (Public health executive)

Respondents valued that the model was transparent about “what’s under the hood”. They noted that they understood the logic of the model and the evidence used to inform it and this increased their trust and willingness to use the model to facilitate policy and program planning conversations.

Model outputs were described as involving a bit of “science and magic” or “computer magic” that was engaging and useful, however concern was expressed that model outputs could be interpreted by people not involved in the participatory process as reality rather than as a decision support tool to compare alternative strategies. Respondents emphasised their awareness of the limitations of the models, the need to ensure that model outputs were interpreted appropriately and for end-users to be aware of the assumptions and limitations of the evidence used in the models.

Many respondents discussed the importance of ensuring that the participant group included representatives for the policy and intervention options being considered in the models. For example, if regulatory interventions were being considered then stakeholders who have regulatory oversight for the issue should be included in the participatory process to increase trust and reduce the risk of resistance from these stakeholders to using the model to support decision making.

#### Using the model to synthesise and facilitate use of evidence in policy conversations

The models were valued as communication tools. They were viewed as giving credibility to the argument for prevention interventions. The ability to manipulate policy levers to switch interventions on and off or to modify the reach and effectiveness of interventions and then observe the impact on outcomes of interest were frequently identified as providing an important evidence base to support policy and planning conversations. For example, model outputs were used to demonstrate the impact of current programs and reinforce with local service providers the importance of maximising reach and effectiveness of their current programs and to inform planning decisions by forecasting the impact of local interventions if scaled up to the population level.

“Well, it's a neat graphical tool that assists the presentation of data on effectiveness of programmes. It's quite neat the way you can say, “Well, if we do programme X, this is the result we're going to achieve on this variable” It’s nice to be able to present a dynamic model like that. In that sense, people engage with it.” (Public health executive)

Dynamic simulation modelling was viewed by most respondents as offering unique benefits, including the ability to investigate the impact of intervention combinations and interactions on outcomes, forecasting delays in intervention effects and therefore informing expectations for performance monitoring of program implementation and providing a tool to test the potential impact of new ‘bold’ innovative interventions before they were rolled out in the real world.

“I think it's the learning, and the benefits of the modelling, and the things it can do that we can't do from normal longitudinal studies or trials, qualitative work, it is impossible to do what the model does, but that's the point of benefit.”(Public health executive)

The ability to dynamically interact with the model to develop insights into which programs needed to be enhanced, which gaps needed to be filled and which target groups to focus on were identified as important benefits derived from dynamic simulation modelling. This dynamic interaction facilitated policy discussions for local health program planning and engagement with other agencies. The model outputs were used to explore which interventions with which target groups would yield bigger benefits in the long term.

Many respondents noted that the interaction with and communication of model insights were areas requiring further development. The development of communication tools, such as interactive user interface tools and presentations that could be easily adapted to different audiences, were viewed as critical to facilitate the use of the models in policy and program discussions to inform decision making.

Due to delays in model development, the intended presentation of one of the models to a broader range of stakeholders was unable to occur within the anticipated project timeline. A small number of respondents identified this as an important missed opportunity from a co-production and participatory research perspective that undermined the application of the model findings to policy decisions.

#### Model maturing process

The dynamic simulation models were commonly viewed as tools that would mature over time. The maturing process was described in terms of continuously refining the inputs, assumptions and parameters used in the model as new knowledge and evidence became available; building on the model when new policy questions arose and maturing the methods used to communicate model outputs such as user interfaces and presentation of results. The allocation of sufficient time to familiarise participants with the use of the model, train “super users” and socialise the models with broader stakeholder groups were identified as important elements to support the communication of model outputs and increase their use in decision making.

Identifying and prioritising gaps in the existing evidence base was an important component of the model maturation process. The interactive discussions regarding the causal factors and impact of interventions for each focus topic facilitated the identification, clarification and prioritisation of gaps in current knowledge and evidence and could be used to guide future research.

## Discussion

The aim of this research was to explore the experience of end-user decision makers who participated in participatory simulation modelling projects and the perceived value and efficacy of this method as an evidence synthesis and decision support method in an applied health sector context from their perspective. Overall, the participatory approach used to develop the dynamic simulation models was valued by these participants, including both senior clinical experts and public health executives, and was an essential process for building trust in the model as a decision support tool. The collaborative, co-production principles used to develop the simulation models facilitated participant understanding of the logic and evidence used in the models and increased their sense of ownership and willingness to use the models for decision making. The models were broadly viewed as useful and convincing communication tools to facilitate policy discussions. The unique benefits of the models included the ability to explore the interaction of risk factors and causal mechanisms; the interaction and combination of public health interventions; the impact of scaling up the reach and effectiveness of existing programs and the impact of new and untested interventions in simulation before they were implemented in the real world. The participatory process was time consuming, changeable and resource intensive. Therefore, complex issues with contested options for intervening were more likely to be viewed as worthwhile for the significant time investment required for participatory model development.

### Motivations for participating

Participating in research activities, including participatory modelling, requires significant time investment for stakeholders and it is important to consider the factors that motivate their participation when planning research [38]. Studies focusing on stakeholder inclusion in health research, have often been from the perspective of the researchers. Factors such as the difficulty of finding stakeholders with the right skills and knowledge who are interested and available to participate or the difficulty of dedicating time to stakeholder engagement in a context where it isn’t measured and may not be valued [39, 40], have been the focus rather than the perspective of the stakeholders and their motivations for becoming involved. The case studies reported here provide insight into the motivations from the stakeholder perspective. Targeted recruitment of stakeholders with a professional involvement and expertise in the topic being modelled, facilitated by a trusted relationship with either the modelling team or a lead domain expert involved in the project were found in these case studies to be important and successful strategies to motivate policy makers to invest their time.

Motivations for community groups to engage in participatory modelling have been found to be highest when the problem needs to be solved with some urgency and existing approaches have already been tried and failed or known to be unsuitable for the problem at hand [16]. In the complex and contested context of the priority focus issues for the case studies reported here, policy makers and health service providers were motivated to engage in dynamic simulation modelling as a new method for evidence synthesis and exploring “what if” scenarios for policy analysis, particularly with a familiar and trusted team.

### Collaboration and co-production were key elements valued in the participatory approach

Relationships and collaborations are frequently identified as critical factors in systems approaches [41, 42]. Participatory dynamic modelling provides a structured process to facilitate inter-disciplinary dialogues and co-production methods involving a range of participants, including end-user decision makers. Participatory modelling approaches aim to combine diverse perspectives to tackle the social complexity of problems and recognise that different types of knowledge contribute alternative and valuable perspectives to the problem discourse [18, 21, 23, 43]. Participants in the case studies reported in this paper viewed the participatory process as a valuable co-production approach to understand the focus issue from a system perspective, for example, enabling the consideration of how decisions made in one part of the health service, or indeed by other government departments, could impact on programs and services in another. The ability to combine the significant knowledge from multiple experts to guide the model development as a decision support tool was viewed as a unique benefit of the participatory process.

Participatory model development and validation has been shown to increase confidence that the model results were both valid and useful for the participants’ local context [19, 27, 44, 45]. Decision maker involvement in model development resulted in them being more likely to draw on the model’s outputs to inform decisions about priority interventions and policies [27, 43, 46]. The involvement of key stakeholders and decision makers in these case studies was identified as critical to developing trust in the use of the model to support decision making. Participants also noted the importance of ensuring that representatives of important stakeholder groups, such as consumers and relevant policy agencies, were included in model development process.

The domain expert and translator roles were identified in these case studies as important to facilitate engagement in the participatory process and use of the model to inform decision making. Similar roles have been identified in community based environmental modelling contexts [16, 45] as playing an important role liaising between the modelling team and stakeholders. The key elements for the translator role include being a member of the stakeholder community who can both identify with the needs and articulate them within a group model building session and has credibility in translating and conveying insights from the modelling process [16]. In the health setting case studies reported here, it was important that the “translator” was embedded within the stakeholder organisation and facilitated the acceptance and use of the model to support policy discussions. Thompson et al. (2010) described the beneficial relationship between the translator and the modellers as the translators driving the modellers to integrate the participants’ requests and insights into the model, and the modellers driving the translators to introduce complex science and dynamic interrelationships to the stakeholders [45].

### Participant learning

Simulation modelling aims to enhance knowledge of participants from the perspective of different disciplines and more thorough exploration of focus problems using systems science [21, 38]. The evaluation framework used to guide the design of this research focused on changes in perceptions about the assumed causality pathways in the system to assess participant learning [35]. Interviewees reported that the participatory process provided opportunity to be exposed to multiple perspectives, interact and contribute expertise, however they did not identify that they had learned about the causal pathways for the focus issue. Participants in the case studies reported here were long term experts in the focus issue, many of whom had dedicated their career to working on the issue, therefore they came to the process with a good understanding of the different perspectives and complex causal relationships and therefore may not have gained new knowledge through the participatory process. However, this finding is consistent with Rouwette et al. who found that participants in group model development processes experienced difficulty identifying their learning from these processes without specific prompting [19].

Participatory modelling processes can be characterised as social learning exercises [27], and the shifts in perceptions and learning that result can play out differently at the individual and group levels [47]. Participatory modelling involves the sharing of knowledge through group discussion, interactive activities and interactions with the model [27, 47]. Recent participatory modelling research in the environmental sciences has found measures of individual cognitive change to be informative, but unable to reflect how the group evolves in their capacity to make decisions informed by the model [47].

All respondents in these case studies reported that they learned about the potential of dynamic simulation modelling to explore complex interrelationships for the focus issue and emergent behaviour of the system to support decision making and the process of developing simulation models through the participatory process. This was identified as an important learning outcome and is consistent with the key benefit of participatory modelling that people learn how to model better, and with better modelling comes better insights to improving the system [16]. To increase the sophistication and effectiveness of participatory modelling facilitation methods, better understanding about how different stakeholder groups evolve the knowledge and skills to work with decision support tools like simulation models to plan policy and programs will be an important area of future research.

### Intentions to use dynamic simulation modelling to inform decision making

Stakeholders are more likely to trust a model if they have been involved in informing and grounding the understanding captured in the model, they understand it and they feel ownership [18, 48, 49]. The participatory approach used in these case studies engaged policy decision makers actively in the model development process which fostered their interest and trust in the model outputs. Making the model understandable and accessible to stakeholders has been identified as an important principle of participatory modelling [49] and was a key benefit commonly identified across these case studies. Participants understood the logic of the model and the evidence used to inform it and this transparency increased their trust and willingness to use the model to facilitate policy and program planning conversations.

Ensuring stakeholder representation for the policy and intervention options being considered in the models was identified as a key consideration for project planning to realise the benefits of the participatory process. Participatory modelling has been shown to successfully facilitate productive problem solving across agency boundaries by providing a neutral platform for discussion and scenario testing to explore a broad range of options and solutions [16, 18, 48, 50]. The participatory process can bring key stakeholders from different agencies responsible for implementing policy and programs together to explore and test “what if” policy scenarios and explore which interventions represent the most effective leverage points in the local context and therefore align and mobilise prevention efforts of community stakeholders [51].

The finalised models in the case studies reported on here are being used as credible, communication tools to synthesise and facilitate use of evidence in policy conversations regarding prevention interventions. The models capture the complexity of real-world policy questions and provide a dynamic analytic tool that can overcome the limitations of traditional analysis methods [52, 53]. The ability to manipulate policy levers to determine impact of interventions on health outcomes (including the ability to test alternative reach, adoption, and effect scenarios) provided an important evidence base to support health policy and planning conversations and develop realistic insights into the impact of enhancing or expanding existing interventions and identify priority gaps and areas of need.

The unique benefits offered by participatory dynamic simulation modelling have previously been identified from the perspective of modelling teams. For example, helping stakeholders understand how multiple variables, factors, and interventions interact, being able to test the potential impact of programs and policies in the “safety” of a virtual environment before they are implemented, saving time, effort, costs and resources, guiding and prioritising data collection and facilitating discussions among stakeholders [49]. The importance of these benefits to end-user decision makers was confirmed by these case studies with participants identifying that the ability to dynamically investigate the impact of intervention combinations and interactions on outcomes, forecast delays in intervention effects and test the potential impact of new innovative interventions before they were rolled out in the real world were valued and utilised in policy and program decision making discussions. The benefits of participatory dynamic simulation modelling methods identified by the policy makers are summarised below. These are benefits that dynamic modelling provides over other forms of knowledge mobilisation [34, 23] from the perspective of end-users of these models as decision support tools.

#### Summary - unique benefits of participatory dynamic simulation modelling identified by policy makers

- Increasing familiarity and trust in the model by use of participatory, co-production methods.
- Synthesising diverse evidence in an interactive and dynamic decision support tool that facilitates the exploration of “what if” scenarios and policy options.
- Exploring the combination and interaction of interventions to develop insights into which interventions to enhance, which gaps to fill and which target groups to focus on
- Exploring the impact of new and untested interventions prior to implementation in real world
- Forecasting delays in intervention effects to guide implementation monitoring
- Identifying and prioritising evidence gaps

### Implementation challenges and future work

The co-production and participatory approaches to model development were time consuming, unpredictable and messy, making them more suitable for longer term planning in the first instance. However, once the models were developed they could be used for short-turnaround policy advice. For these case studies, the intended presentation of one of the finished models to a broader range of stakeholders did not occur due to timing and resource constraints. This was as an important missed opportunity from a co-production and participatory research perspective that undermined the application of the model findings to policy decisions.

The refinement of communication tools, such as interactive user interface tools and presentations that could be easily adapted to different audiences, will be critical to facilitate the use of the models in policy and program discussions to inform decision making.

Supporting good understanding of the model development process, for example, how decisions are made regarding the methods used to represent causal mechanisms dynamically, where to add complexity, where to simplify the model and how to deal with and communicate uncertainty in the models are important ongoing challenges. These are the subject of future work in this program of research on participatory modelling.

The dynamic simulation models are tools that mature over time with the inputs, assumptions and parameters being continuously refined and updated as new knowledge and evidence become available. The identification and prioritisation of gaps in the existing evidence base was facilitated by the interactive discussions regarding the causal factors and impact of interventions for each focus topic and used to guide future research priorities.

Key implementation strategies are summarised below. The strategies cover practical aspects of the workshop facilitation and important aspects of communication and engagement with participants before, during and after the model development workshops. It is important to acknowledge that these strategies are not intended to be prescriptive. Each modelling has project has unique requirements, stakeholders and policy context, and therefore being flexible and responsive to project needs and stakeholder feedback is critical.

#### Summary of implementation strategies for project phases

#### Key project roles

*Domain expert* **–** well-respected authority on the focus issue who can play a lead role in the project planning and workshop facilitation.

*Translator* **–** person who can contextualise the policy environment and data for the modelling team and translate the model requirements and development process to the participants.

*Expert participants* **–** people with a range of expertise, including providing or planning health services, undertaking research or developing policy for the issue in focus.

*Dynamic simulation modeller* – person with computer programming and data analysis expertise in developing dynamic simulation models.

*Super-user –* person who learns to use the model interface and apply it to explore policy scenarios “in-house”. They are usually employed by the jurisdictional health department in analytic roles.

#### Planning phase

1. Use a domain expert to facilitate engagement and trust.
2. Engage a broad range of participants to provide diverse and representative perspectives. Important to include “domain expert”, “translator”, “clinical experts”, “modeler”, “policymakers”, “super users”
3. Provide background briefing material about the participatory process prior to the workshops to enable participants to prepare and do “pre-thinking”
4. Increase motivation to participate by engaging with key stakeholders from lead agencies in the planning phase to ensure the focus topic is high priority and of professional interest. Complex issues with contested options for intervening are more likely to be viewed as worthwhile for the significant time investment required for participatory model development.
5. Wherever possible, book workshops and meetings well in advance to provide the best opportunity for a broad range of stakeholders to be able to attend.

#### Participatory model development phase

1. Use “translators” to facilitate the communication of technical concepts between the content experts and the modelling team.
2. Use intuitive and engaging activities and ensure that these are sufficiently prepared in advance. The activities used in these modelling case studies are described elsewhere [34].
3. Prioritise opportunities for participants to actively engage and interact with each other and hands-on model development activities over less interactive update sessions. Provide opportunities to have fulsome discussions about priority issues.
4. Have a clear agenda and keep to time, as much as possible, while maintaining flexibility to allow important discussions to continue or to move on from activities that are completed.
5. Use small group work where possible to increase active participation.
6. Choose venues with sufficient physical space and technical capacity.

#### Communication outside workshops

1. Maintain frequent communication with participants providing progress reports, answering questions, requesting advice and evidence.
2. Provide opportunities for direct interaction between key participants and the modelling team to refine the model scope and direction.
3. Identify where key issues remain for participants and work together to try and resolve them e.g. refining the definitions of categories or parameters used in the model.

#### Using the model to inform decision making

1. Be transparent about the logic, assumptions and parameters used in the model.
2. Use “translators” to facilitate ongoing interaction with the model and communication of model outputs to stakeholders.
3. Ensure that time is provided to socialise the model with a broader stakeholder group who were not involved in the participatory process.
4. Develop simple, clear and concise key messages about insights from the model
5. Develop associated tools to facilitate communication e.g. an intuitive and interactive user interface and adaptable presentations to suit a variety of audiences.

### Limitations

Two of the three models developed in these case studies were finalised at the time of the interviews. The participant perspectives of the utility of participatory dynamic simulation to inform decision making was thus limited to these two case studies. The perspectives included this analysis were limited to participants employed by government agencies with jurisdiction over many of the policy and program decisions relevant to the focus topics being modelled in these case studies. Their perspectives may vary from those of other participants in the case studies, for example academic researchers or representatives from non-government agencies. Decision maker’s views of different methods of dynamic simulation modelling, e.g. system dynamics vs agent based modelling, will be an important issue to explore in future research as decision makers develop and broaden their experience of different forms of modelling.

The lack of consumer representation in these case studies is a limitation. The development of strategies to realise the benefits of consumer involvement in participatory dynamic simulation modelling to inform health policy decisions is an important area for future focus.

## Conclusion

The case studies reported here have provided new insights into the experience of engaging in participatory dynamic simulation modelling from the perspectives of the end-user policy makers and senior clinicians with decision making roles in Australian jurisdictional health departments. The participatory, co-production process was viewed as an essential approach to ensure the dynamic simulation models incorporated the best available knowledge and evidence for the focus issue and that the models were well understood or “transparent” to build trust in the model as a decision support tool for policy discussions. The unique benefits of the dynamic simulation models included being able to synthesise diverse evidence; explore the combination and interaction of risk factors and interventions; explore the impact of new and untested interventions in silico; and identify evidence gaps to prioritise for future research. Given the commitment of time and resources to the participatory model development process, it was important to ensure that the topic justified the investment i.e. It was a high local priority, complex and had contested options for intervening. Engaging domain experts and people to work as “translators” from within the stakeholder organisation were important to facilitate engagement in the process and use of the models as policy decision support tools. Participatory modelling processes are more suitable for longer term planning in the first instance (prior to the model being developed), however are responsive to short-turnaround policy advice once developed as a decision support tool. The ongoing refinement of model development workshop activities and communication tools to support the application of model findings to policy decisions will be important foci for future research on these methods.

## Additional file

Additional file 1: Indicative questions for pre-modelling workshop interviews. Indicative questions for post-modelling semi structured interviews. Interview scripts and questions. (DOCX 16 kb)
