# Supplementary material for: Decision makers’ experience of participatory dynamic simulation modelling: methods for public health policy
Source: BMC Med Inform Decis Mak. 2018 Dec 12;18:131. doi: 10.1186/s12911-018-0707-6 (PMC6291959; doi:10.1186/s12911-018-0707-6)
Supplement: Supplementary file 1 — Indicative questions for pre-modelling workshop interviews. Indicative questions for post-modelling semi structured interviews. Interview scripts and questions. (DOCX 16 kb) [file 12911_2018_707_MOESM1_ESM.docx]

## Appendix A – Indicative questions for semi-structured interviews

### Indicative questions for pre-modelling workshop interviews

*Introduction*

*Thank you very much for agreeing to be interviewed for this project. I am going to ask you some questions about challenges that gestational diabetes health services are facing, about your experience of evidence based decision and making and your experience with simulation modelling processes.*

***GESTATIONAL DIABETES SERVICES***

1. Based on your experience, what are the current challenges that GDM services are facing?
2. What do you think is driving these challenges?
3. What changes do you think GDM services need to make to cope with these challenges?
4. Which interventions would you prioritise to prevent and manage GDM?

***EVIDENCE BASED DECISION MAKING***

*The next questions are about evidence-based decision making.*

1. Could you talk a little about your thoughts on evidence-based decision making in the health policy context?
2. What factors have you found to be useful to support its use? What are the main challenges?
3. Have you had experience using results of evidence synthesis methods such as systematic reviews, meta analyses? Did they meet your needs for evidence to inform your decision making? From your experience, what are the strengths and limitations of these methods?
4. What other forms of evidence do you use in decision making?

***SIMULATION MODELING***

*The following questions are about any experience you have had with simulation modeling processes*

1. Have you participated in any form of dynamic simulation modeling process before? *(Only continue if reply yes)*
2. Could you tell me about the modelling process and your experience of it?
3. In your opinion what are the benefits and limitations of simulation modelling as an evidence synthesis tool?

***FINAL QUESTION***

*Finally, we are interested in your goals for participating in the project.*

What do you hope to get out of participating in this modeling process?

*Thank you very much for agreeing to be interviewed. Just before we finish, do you have any questions that you would like to ask about this project?*

### Indicative questions for post modelling semi-structured interviews.

Introduction:

·     I am interested in hearing about your experience of participating in the XX dynamic simulation modelling project.

·     We have several projects that have used similar methodology and are at different stages of maturity.  I would like to talk to you about your experiences with the XX modelling projects so we can collect information on impact in different settings and at different stages of maturity

·     I am keen to hear your honest appraisal of the pros and cons of this method

1.     How did you come to be involved in the project? When you were first approached, what were your thoughts? Why did you agree? What were your expectations at the beginning of the modelling project? Were those expectations met?

2.     Were there aspects of the workshops that you found useful? [Prompt as needed - What were these and why?]

3.     I’d like to ask you a bit about some of the different aspects of the participatory process. Could you tell me about your experience of:

a.     The activities at the workshops

b.     The interactions with professionals from a range of disciplines

c.     The model outputs

(prompt as needed – what do you feel you gained from these)

4.     We are interested in exploring the value of using a participatory approach to develop the model. In your view, what were the benefits of using this approach? And what were the challenges?

5.     One key aspect of group model building is to bring together a diverse group of experts to discuss and compare their “mental models” (by that I mean their individual understanding of an issue and its context).  The aim of this is to enable the modellers to learn about the issue from a range of perspectives, and for the expert participants to compare their own perspectives to that of others in the group. What are your thoughts about this aspect of the workshops? In your opinion, how successful were the workshops in enabling this to occur?

6.     Having been through the experience of the workshops, what are your thoughts now on how dynamic modelling can facilitate evidence being used to inform decision making?

 7.     Have you been able to apply any insights gained from the modelling process to your work? If so, could you talk about some examples? In your opinion, were your insights based on your involvement in the workshops or from the results generated by the model?

8.     Could you talk a bit about the purpose for which the model was developed and the broader context? I’m interested to hear whether this has had any influence on its subsequent use.

9.     Since you participated in the modelling process, have you been able to use the model outcomes or insights you gained from the process to build an argument for prevention programs?

10.     Would you like to see this work developing into the future? Would you be interested in being involved?
